# IL-33-ST2 Axis in Liver Disease: Progression and Challenge

**DOI:** 10.1155/2017/5314213

**Published:** 2017-10-18

**Authors:** Zijian Sun, Binxia Chang, Miaomiao Gao, Jiyuan Zhang, Zhengsheng Zou

**Affiliations:** ^1^Center of Non-Infectious Liver Diseases, Peking University 302 Clinical Medical School, Beijing, China; ^2^Center of Non-Infectious Liver Diseases, Beijing 302 Hospital, Beijing, China; ^3^Treatment and Research Center for Infectious Diseases, Beijing 302 Hospital, Beijing, China

## Abstract

The new member of the IL-1 family, interleukin-33 (IL-33), participates in the progression of a variety of diseases through binding with its receptor ST2. Recently, much clinical evidence and experimental data have indicated that IL-33 is associated with various liver diseases. This review primarily addresses the relationship between IL-33 and several hepatic diseases. IL-33 can alleviate high-fat diet- (HFD-) induced hepatic steatosis and insulin resistance, and IL-33 acts as an alarmin, which quickly triggers the immune system to respond to virus invasion and toxic damage to the liver. However, when liver injury is chronic, IL-33 promotes Th2 reactions and hepatic stellate cell (HSC) activity, facilitating progression to liver fibrosis. The complicated functions of IL-33 should be considered before its clinical application.

## 1. Brief Introduction of IL-33

Interleukin-33 (IL-33) was originally discovered (as clone DVS 27) in a study of canine vasospastic cerebral arteries after subarachnoid haemorrhage and received attention due to its highly upregulated expression [1]. IL-33 was then identified as NF-HEV expressed in human high endothelial venules and shown to function as a nuclear factor [2]. In 2005, Schmitz and coworkers matched the sequence structure of IL-33 with the IL-1 cytokine family, and IL-33 was successfully classified as part of the IL-1 family and named IL-33. The IL-33 gene is located on chromosome 9p24.1 in humans and on the syntenic chromosome 19qC1 region in mice. IL-33 cDNA encodes 270 and 266 amino acid polypeptides in humans and mice, respectively, and the full-length proteins have respective masses of 30 and 29.9 kDa. Caspase-1 can cleave IL-33 to a mass of 18 kDa, and the amino acid similarity between human and mouse IL-33 is up to 55% [3]. IL-33 mRNA can be found in multiple cells and tissues in both humans and mice; at the protein level, IL-33 is broadly expressed in endothelial cells, epithelial cells, smooth muscle cells, and several organs, such as the lung and central nervous system [4–6].

The excellent work by Schmitz et al. indicated that IL-33 is the ligand of the orphan receptor ST2, which is a member of the IL-1 receptor family [3]. ST2 protein exists in at least 3 isoforms through diverse splicing: a transmembrane form (ST2L), a soluble form (sST2), and a novel variant [7]. ST2 is expressed by several types of haematopoietic cells [3]. In addition, ST2 is selectively and stably expressed on the surface of T helper 2 (T_H_2) cells but not on that of T_H_1 or regulatory T (Treg) cells [8, 9]. IL-33 binds ST2, which then connects with IL-1R accessory protein (IL-1RcAP) to form a heterodimeric complex [10]. sST2 is considered a decoy receptor [11] that competes with ST2L for IL-33 binding, subsequently blocking the IL-33 signalling pathway [12]. Single immunoglobulin IL-1R-related molecule (SIGIRR) associated with ST2 has the ability to inhibit IL-33/ST2-mediated signalling [13].

Binding of IL-33 with its receptor recruits myeloid differentiation primary response protein 88 (myD88), IL-1R-associated kinase 1 (IRAK1), and IRAK4 to the receptor through IL-1RAcP's Toll-interleukin 1 receptor (TIR) domain [3]. Subsequently, certain downstream signalling molecules are activated, including nuclear factor-*κ*B (NF-*κ*B) [14], inhibitor of NF-*κ*B-*α* (I*κ*B*α*), extracellular signal-regulated kinase (ERK), p38, serine/threonine protein kinase Akt (protein kinase B), and c-Jun N-terminal kinase (JNK) [15].

Based on the conserved homeodomain-like helix-turn-helix motif in the N-terminal portion of IL-33 and on the nuclear localization signal, IL-33 may play dual roles. First, it behaves as a traditional cytokine activating downstream signalling, and second, it acts as an intracellular nuclear factor with transcriptional regulatory properties [16]. Among the numerous biological functions of IL-33, the best known is promoting polarization of naïve T cells to T_H_2-type immune response cells, and it can act directly on T_H_2 cells to increase IL-5 and IL-13 production [3]. Furthermore, IL-33 has been identified as a selective Th2 chemoattractant [17]. In addition to T_H_2 cells, IL-33 also interacts with many other immune cells. For example, IL-33 attenuates TLR4-induced downregulation of CXCR2 and chemotaxis in neutrophils [18], and IL-33/ST2 signalling participates in alternatively activated M2 macrophage polarization in macrophages [19]. Another well-known function of IL-33 is that of an “alarmin”; under cell damage (necrosis) or mechanical injury conditions, active full-length IL-33 can be released rapidly to alter the immune system response [20].

IL-33 is associated with a variety of diseases. Whether IL-33 promotes or inhibits disease progression depends on the type of disease. In asthma patients, IL-33 expression was elevated significantly [5, 21], and in a mouse model of asthma, ST2^−/−^ mice developed attenuated airway inflammation [22]. IL-33 levels were elevated in sera and synovial fluid samples of rheumatoid arthritis (RA) patients and were correlated with the activity of the disease [23]. The serum level of IL-33 decreased after anti-TNF treatment and was correlated with the production of IgM and RA-related autoantibodies [24]. IL-33 expression was significantly increased in the inflamed mucosa of inflammatory bowel disease (IBD) patients as well as in colitis mice induced by dextran sulphate sodium (DSS) [25, 26]. IL-33 expression in the brain was downregulated in Alzheimer's disease (AD) cases compared with controls [27], and a similar conclusion was drawn by another study [28]. Recently, it has been reported that IL-33 can ameliorate AD-like pathology and cognitive decline, and the authors proposed that IL-33 is a promising potential treatment for AD [29]. As the IL-33 decoy receptor, the sST2 level rose immediately after acute myocardial infarction [30], and the serum ST2 level was identified as a novel biomarker for neurohormonal activation in heart failure patients [31]. On the other hand, researchers have found that in ischemia/reperfusion model rats, IL-33 prevented cardiomyocyte apoptosis and enhanced cardiac function through ST2 signalling [32].

## 2. IL-33 and Liver Disease

The relationship between IL-33 and liver disease, as well as its role in the development of liver disease, has attracted the attention of an increasing number of researchers. So far, IL-33 has been found to be involved in a variety of liver diseases, including fatty liver disease, hepatitis, liver fibrosis, and cirrhosis, along with other hepatic diseases (Table 1).

### 2.1. IL-33 and Fatty Liver Disease

As recently as 20 years ago, researchers realized that in many industrialized countries, nearly a quarter of adults had excessive fat accumulation in the liver, and fatty liver was a vital risk factor for serious liver disease [33]. Studies regarding the role of IL-33 in fatty liver disease have primarily focused on nonalcoholic fatty liver disease (NAFLD). The spectrum of NAFLD ranges from fatty liver alone to nonalcoholic steatohepatitis (NASH), which may progress to cirrhosis and its associated complications without a history of heavy alcohol consumption [34, 35]. NAFLD is commonly found in type 2 diabetes and obese patients, and insulin resistance is closely related to NAFLD development and prognosis [36]. One study showed that *in vitro* administration of IL-33 into adipose tissue cultures induced Th2 cytokine production (IL-5, IL-13, and IL-10) and downregulated the expression of adipogenic genes. Administration of IL-33 to genetically obese diabetic (ob/ob) mice resulted in reduced adiposity and improved glucose and insulin tolerance [37].

Because of the regulatory role played by IL-33 in lipid metabolism, IL-33 may have a close relationship with fatty liver. An NAFLD mouse model was successfully constructed by feeding mice with a high-fat diet (HFD) [38]. The results of a recent study showed that a HFD given to mice for 20 weeks induced upregulation of both IL-33 and ST2 mRNA and proteins. Furthermore, treatment with IL-33 alleviated HFD-induced hepatic steatosis, reduced serum alanine aminotransferase (ALT) levels, and ameliorated insulin resistance and glucose intolerance. Notably, the researchers found that the serum IL-33 levels and IL-33 mRNA levels in the liver were higher in NAFLD patients. The group also confirmed that IL-33 promoted a Th2 response, M2 macrophage activation, and fatty acid metabolism gene expression in the liver [39]. Meanwhile, in another study, ST2^−/−^ mice fed with a HFD exhibited increased weight gain and visceral adipose tissue, but the deletion of ST2 ameliorated hepatic steatosis and inflammation [40]. Because the IL-33/ST2 axis may have a disputably beneficial effect on fatty liver, more studies are needed to clarify its mechanism and determine its therapeutic value.

### 2.2. IL-33 and Hepatitis

Multiple aetiological factors can lead to hepatitis, such as viral infection, alcohol abuse, toxicants, drugs, and autoimmunity. Changes in IL-33 expression in viral hepatitis and fulminant hepatitis triggered by toxins have been recently reported, suggesting that IL-33 may participate in different types of hepatitis. Approximately 70% of hepatitis C virus- (HCV-) infected patients cannot completely clear the HCV and eventually can develop persistent chronic infection. Cirrhosis and hepatocellular carcinoma can develop in many of these patients [41, 42]. What is the role of IL-33 in this disease? Wang et al. drew conclusions by comparing chronic hepatitis C (CHC) patients, spontaneously resolved HCV (SR-HCV) patients, and healthy controls (HCs). They found that serum IL-33 levels in CHC patients were significantly higher than those in HC and SR-HCV patients, while IL-33 levels decreased after treatment with interferon for 12 weeks, and this decrease was correlated with ALT and aspartate aminotransferase (AST) levels in CHC patients [43]. Meanwhile, Hamdi and coworkers obtained similar results [44]. In another study, serum HCV RNA was also detected, and it was found that serum IL-33 concentrations were positively correlated with the levels of serum HCV RNA [45].

As a pathogenic factor, IL-33 plays a role not only in CHC but also in chronic hepatitis B (CHB). Hepatitis B virus (HBV) is another major cause of chronic liver disease. After HBV infection, most adults can clear the virus spontaneously, but nearly 5% of infected adults and more than 90% of infected infants and young children will develop chronic infection [46]. CHB also has a risk of progressing to liver cirrhosis and hepatocellular carcinoma [41, 42]. One study showed that the serum IL-33 and ST2 levels were elevated as serum ALT concentrations increased in CHB patients compared to HBV carriers, HCs, and CHB patients with low ALT levels [47]. All these results suggest that IL-33 is associated with liver damage. Therefore, IL-33 has been proposed to function as an alarmin to alert the immune system of tissue damage following infection.

To facilitate research, many researchers have explored the relationship between IL-33 and hepatitis in animal hepatitis models. Arshad et al. detected IL-33 expression in murine fulminant hepatitis induced by poly(I:C), a Toll-like receptor (TLR3) viral mimetic, and by pathogenic mouse hepatitis virus (L2-MHV3). Their results showed that in both hepatitis mouse models, the expression of IL-33 was upregulated and hepatocyte-specific IL-33 expression was downregulated in natural killer cell- (NK-) depleted poly(I:C)-treated mice with severe liver injury, while natural killer T cell- (NKT-) deficient mice exhibited hepatoprotection against poly(I:C)-induced hepatitis accompanied by an increased number of IL-33-expressing hepatocytes compared with wild-type (WT) controls [48]. Lymphocytic choriomeningitis virus- (LCMV-) infected IL-33-knockout mice were used in another study, and the study indicated that IL-33 deficiency resulted in fewer IFN-*γ*^+^* γδ* T and NK cells. In contrast, recombinant IL-33 (rIL-33) facilitated IFN-*γ*-producing *γδ* T and NK cells and inhibited IL-17^+^* γδ* T cells, revealing a role of IL-33 in regulating innate IFN-*γ* production and antiviral responses in LCMV-infected hepatitis [49, 50]. Liang et al. used another virus, adenovirus (Ad), to induce hepatitis. During the first week of Ad infection, a continuous increase in IL-33 and ST2 expression was observed in mouse livers. Injection of rIL-33 resulted in a decrease in serum ALT levels and the number of Councilman bodies in the liver. These changes were correlated with the upregulation of Treg cells and downregulation of macrophages, dendritic cells, and NK cells in the liver, and at the same time, TNF-*α* expression was inhibited by IL-33 in hepatic T cells and macrophages, and TNF-*α* levels in the liver decreased [51].

Another focus of the relationship between IL-33 and hepatitis is the protective role of the IL-33/ST2 axis in concanavalin A- (Con A-) induced hepatitis. A study researched by Volarevic and coworkers indicated that severe hepatitis developed in Con A-treated ST2-deficient mice, and these mice exhibited a high number of mononuclear cells in the liver and a high level of proinflammatory cytokines (TNF-*α* and IFN-*γ*). In contrast, in WT mice, the number of CD4^+^Foxp3^+^ cells was statistically higher. Furthermore, injection of IL-33 into WT mice attenuated liver damage and increased the number of liver CD4^+^Foxp3^+^ cells. IL-33 also suppressed caspase-3 activation and the expression of BAX and enhanced Bcl-2 expression in the liver [52]. Interestingly, NKT-deficient mice were also resistant to Con A-induced hepatitis and no longer expressed IL-33 in liver cells following Con A administration, while IL-33 was overexpressed in normal mice [53]. Meanwhile, IL-33 expression in hepatocytes was also blocked during Con A-induced acute hepatitis in tumour necrosis factor-related apoptosis-inducing ligand- (TRAIL-) deficient mice, and IL-33-deficient mice exhibited more severe Con A-induced liver injury than WT mice [54]. Furthermore, the severity of liver injury in IL-33^−/−^ mice was positively correlated with TNF-*α* and IL-1*β* levels and the number of NK cells infiltrating into the liver [55]. The majority of studies in this area support the view that IL-33 protects against Con A-induced hepatitis, and this protection involves a variety of immune cells (Treg, NK, and NKT cells) and molecules (IFN-*γ* and TRAIL). However, the opposite result was obtained in one study: treatment of rIL-33 exacerbated Con A-induced hepatitis, but pretreatment with an IL-33-blocking antibody exhibited a protective effect, likely by suppressing the late stage of T cell and NKT cell activation and decreasing IFN-*γ* production [56]. More studies are needed to determine whether IL-33 protects or aggravates hepatitis induced by drugs and to elucidate the reasons for this discrepancy.

### 2.3. IL-33 and Liver Fibrosis (and Cirrhosis)

Liver fibrosis and its end-stage form, cirrhosis, are the common final pathway for virtually all chronic liver diseases. Accumulation of extracellular matrix (ECM) rich in fibrillar collagens (mainly collagen I and collagen III) is the characteristic of advanced fibrosis, and it is associated with liver failure, portal hypertension, and a high risk of liver cancer [57, 58]. In the course of chronic hepatitis and progression to cirrhosis, in addition to persistent inflammatory infiltrate, a Th2-polarized immune response always occurs. Th1 cytokines lead to a rapid and intense inflammatory response while causing little fibrosis. In contrast, Th2 cytokines, such as IL-13, promote hepatic stellate cell (HSC) proliferation, transforming growth factor-*β* (TGF-*β*) synthesis, and fibrogenesis [59].

Based on the crucial role of Th2 cytokines in liver fibrosis formation and the pro-Th2 activity of IL-33, the relationship between IL-33 and liver fibrosis has received much attention. One study has shown that in mouse and human fibrotic livers, IL-33 and ST2 mRNA is overexpressed. Moreover, IL-33 expression was correlated with collagen expression, and the major source of IL-33 in fibrotic livers was HSCs [60]. Another study deeply explored the mechanism of IL-33 in promoting the pathogenesis of hepatic fibrosis; this mechanism involved a new type of lymphocyte, innate lymphoid cell type 2 (ILC2), which expresses IL33R-ST2. IL-33-responsive ILC2 cells are widely distributed in the mesenteric lymph nodes, spleen, and liver of mice and produce several Th2 cytokines, such as IL-4, IL-5, and IL-13 [61, 62]. The study also revealed that in hepatic fibrosis, IL-33 expression was elevated, and excess ECM deposition was sufficiently driven by IL-33 alone in the liver. Furthermore, IL-33^−/−^ mice displayed a significant amelioration of experimental fibrosis. IL-33 led to activation and accumulation of ILC2 cells through ST2 signalling in the liver. Activated ILC2 cells produced IL-13, and then, IL-13 initiated activation and differentiation of HSCs via the IL-4R*α*-STAT6 transcription factor-dependent pathway [63]. Meanwhile, another study showed that activation of HSCs was decreased in ST2-deficient liver fibrosis mice and that HSCs were activated by rIL-33 *in vitro*, releasing IL-6, TGF-*β*, *α*-SMA, and collagen [64].

Although earlier we introduced the idea that IL-33 could alleviate HFD-induced hepatic steatosis and insulin resistance, it has been verified that in diet-induced NASH, IL-33-mediated aggravation of hepatic fibrosis was dependent on the ST2 signalling pathway [39]. In primary biliary cirrhosis (PBC), an autoimmune liver disease with complications such as cirrhosis, liver failure, and hepatoma carcinoma [65], the serum IL-33 level of patients was positively correlated with severity [66]. In general, IL-33 showed a potential promotive effect on liver fibrosis.

There are also research teams studying the role of IL-33 in other liver diseases; for example, oncogenesis and progression of hepatocellular carcinoma (HCC) are associated with aberrant IL-33 expression [67], and upregulation of IL-33, primarily produced by CD8^+^ CD62L^−^ KLRG1^+^ CD107a^+^ T cells, may indicate prolonged patient survival [68]. However, high levels of serum sST2 were considered a negative HCC prognostic factor [69]. During *Schistosoma japonicum* infection, IL-33 participated in hepatic granuloma pathology [70]; in the liver of *Leishmania donovani*-infected mice, the IL-33/ST2 axis suppressed Th1 response, and patients with visceral leishmaniasis exhibited higher serum IL-33 levels [71]. IL-33 exhibited a significant protective effect on a liver ischemia/reperfusion mouse model and attenuated liver damage and limited inflammatory activity [72, 73].

## 3. Conclusion and Expectations

It has been nearly 30 years since the discovery of IL-33, and many studies have been performed to determine the molecular structure, distribution, receptor, and signalling pathway of IL-33. Knowledge regarding the molecular basis of IL-33 signalling is relatively comprehensive. Nevertheless, the role of IL-33 is different in various liver diseases. IL-33 can attenuate hepatic steatosis and act as an alarmin by quickly triggering the immune system to respond to virus invasion and toxicant-induced damage, thus leading to a protective effect on viral hepatitis and Con A-induced liver injury. However, IL-33 promotes Th2 reactions and HSC activity, facilitating the progression to liver fibrosis. Therefore, evidence suggests that when acute and massive liver damage occurs, the release of IL-33 by injured hepatocytes might be a protective mechanism, while in chronic injury, IL-33 plays the role of a hepatic fibrosis-enhancing factor. Thus, it is necessary to judge and weigh the opposing functions of IL-33 before clinical application [74].

Although great progress has been made in understanding the relationship between IL-33 and liver disease, the majority of studies are still based on correlations between IL-33 expression and liver disease. Studies on the specific mechanism are not thorough or sufficiently comprehensive, and several experimental results suggest opposite conclusions. Hence, more studies are required to fully understand the role of IL-33 in the regulation of liver disease and its signalling pathways and regulatory networks.

Moreover, we also hope to expand the study of IL-33 to more liver diseases and find more potential therapeutic applications of IL-33. For instance, alcoholic liver disease (ALD) exhibits a disease progression similar to that of NAFLD (from simple fatty liver to alcoholic hepatitis, cirrhosis, and even HCC), and its incidence is rapidly increasing. ALD is becoming an important cause of chronic liver disease worldwide, while (except for abstinence) ALD lacks therapeutic drugs with definite efficacy [75]. Therefore, it is very likely that the role of IL-33 in ALD will be discovered and provide a new treatment approach for ALD. Further study of IL-33, a novel cytokine, could establish a new field of research on the mechanisms and treatment of liver disease.

## Figures and Tables

**Table 1:** Studies on the roles of IL-33 and ST2 in liver diseases.

Disease	Result	Ref
Fatty liver disease	(i) The mRNA and protein levels of both IL-33 and ST2 were increased in the mouse model of HFD-induced hepatic steatosis, and treatment with IL-33 alleviated hepatic steatosis. (ii) ST2^−/−^ mice fed with HFD exhibited increased weight gain, severe hepatic steatosis, and inflammation. (iii) The IL-33 mRNA levels in serum and liver were increased in NAFLD patients.	[39] [40] [39]

Hepatitis	(i) Serum IL-33 levels in CHC patients were significantly higher than those in HCs while decreased after treatment with interferon and were correlated with the ALT and AST concentrations. (ii) Serum IL-33 concentrations in CHC patients were positively correlated with the levels of serum HCV RNA. (iii) CHB patients with high serum ALT concentrations showed higher serum IL-33 and ST2 levels. (iv) In poly(I:C)-induced murine fulminant hepatitis, the expression of IL-33 was upregulated, and in NK-depleted poly(I:C)-treated mice, liver injury was severe while NKT-deficient mice showed hepatoprotection against poly(I:C)-induced hepatitis accompanied by an increased number of IL-33-expressing hepatocytes. (v) IL-33-knockout mice infected by LCMV produced fewer IFN-*γ*^+^* γδ* T and NK cells, and rIL-33 treatment facilitated IFN-γ-producing *γδ* T and NK cells and inhibited IL-17^+^* γδ* T cells. (vi) IL-33 and ST2 levels were increased in mouse liver after Ad infection. Injection of rIL-33 resulted in a decrease in serum ALT levels and the number of Councilman bodies in the liver; meanwhile, Treg cells were upregulated and TNF-*α* levels in the liver decreased. (vii) ST2-deficient mice developed severer hepatitis induced by Con A with a higher number of mononuclear cells and higher level of proinflammatory cytokines in the liver. IL-33 also suppressed caspase-3 activation and BAX expression as well as enhanced Bcl-2 expression in the liver. (viii) NKT-deficient mice performed resistant to Con A-induced hepatitis and lacked IL-33 expression in liver cells. (ix) IL-33 expression in hepatocytes was blocked during Con A-induced acute hepatitis in TRAIL-deficient mice. (x) The severity of liver injury in IL-33^−/−^ mice was positively correlated with the levels of TNF-*α* and IL-1*β* and the number of NK cells infiltrating into the liver. (xi) rIL-33 exacerbated Con A-induced hepatitis, while pretreatment of an IL-33-blocking antibody exhibited a protective effect.	[43, 44] [45] [47] [48] [49, 50] [51] [52] [53] [54] [55] [56]

Liver fibrosis/cirrhosis	(i) In mouse and human fibrotic livers, IL-33 and ST2 mRNA was overexpressed and the major sources of IL-33 were HSCs. (ii) IL-33 led to activation and accumulation of ILC2 through ST2 signalling in the liver, and activated ILC2 produced IL-13; then, IL-13 initiated activation and differentiation of HSCs. (iii) In ST2-deficient mice with liver fibrosis, the activation of HSCs was decreased and *in vitro* HSCs activated by rIL-33 release IL-6, TGF-*β*, *α*-SMA, and collagen. (iv) Serum IL-33 levels of PBC patients were positively correlated with the severity of PBC.	[60] [63] [64] [66]

Others	(i) A high level of IL-33 mainly produced by CD8^+^ CD62L^−^ KLRG1^+^ CD107a^+^ T cells might indicate prolonged patient survival. (ii) A high level of serum sST2 is regarded as a negative HCC prognostic factor. (iii) IL-33 presented a significant protective effect on liver ischemia/reperfusion mouse model with attenuated liver damage and limited inflammatory activity. (iv) IL-33 participated in hepatic granuloma pathology during *Schistosoma japonicum* infection. (v) In *Leishmania donovani*-infected liver mice, the IL-33/ST2 axis suppressed Th1 response and patients with visceral leishmaniasis showed higher serum IL-33 levels.	[68] [69] [72, 73] [70] [71]
